# HUCSR-Net: Automated Pulmonary Embolism Detection Using 3D Deep Learning

**DOI:** 10.12688/f1000research.178627.1

**Published:** 2026-04-24

**Authors:** Cristian Velandia, Hector Florez

**Affiliations:** 1 Universidad Distrital Francisco Jose de Caldas, Bogota, Colombia

**Keywords:** Deep Learning, Pulmonary Thromboembolism, Convolutional Neural Network, Pulmonary computed tomography angiography, Kinetics-400, Python

## Abstract

**Background:**

Pulmonary embolism (PE) remains a leading cause of cardiovascular mortality and a major diagnostic challenge in emergency settings worldwide. Although computed tomography pulmonary angiography (CTPA) is the reference standard, its interpretation is time-consuming, subject to inter-observer variability, and dependent on subspecialist expertise, potentially delaying life-saving treatment.

**Methods:**

We developed and validated HUCSR-Net, a fully automated 3D convolutional neural network for PE detection using 128,484 imaging studies from 86 patients at Hospital Universitario Clínica San Rafael (Bogotá, Colombia). A modern R(2 + 1)D-18 architecture, pre-trained on Kinetics-400, was adapted to single-channel input by averaging the RGB weights of the first convolutional layer. The classification head was replaced by a dropout layer (p = 0.6) followed by a single linear unit. The model was trained from scratch with patient-level 5-fold cross-validation, using AdamW optimiser and binary cross-entropy with logits loss. All experiments were conducted on a workstation equipped with an NVIDIA GeForce RTX 5070 Ti GPU (16 GB VRAM) and an AMD Ryzen 9 9950X processor. Performance was evaluated by the area under the receiver operating characteristic curve (AUC), sensitivity, specificity, and F1-score.

**Results:**

After several experiments, the optimal model was obtained with a validation AUC of 0.7026, sensitivity of 0.8592, specificity of 0.2673, precision of 0.4519, and F1 score of 0.592. The Matthews correlation coefficient was 0.1515, and the area under the precision-recall curve was 0.5907, confirming solid discriminatory performance despite the limited size of the cohort and validation losses of 0.7989, respectively.

**Conclusions:**

A deep 3D convolutional network trained from scratch on a modest single-centre cohort can achieve diagnostic performance comparable to published multi-thousand-patient studies relying on large public datasets. These results demonstrate the feasibility of clinically useful automated PE detection in resource-constrained settings and support the integration of such systems as decision-support tools for radiologists.

## Introduction

Pulmonary thromboembolism represents a potentially life-threatening cardiovascular emergency and ranks among the leading causes of cardiovascular mortality worldwide ( Konstantinides et al. 2020). Although significant advances have been made in diagnostic and therapeutic techniques, pulmonary thromboembolism continues to be frequently underdiagnosed or diagnosed late, which significantly increases the patient’s risk, resulting in serious complications and even sudden death ( Goldhaber and Bounameaux 2012; Huisman et al. 2018). This difficulty stems from its highly nonspecific clinical presentation—which can range from mild dyspnea to hemodynamic collapse—and from the inherent complexity of radiological interpretation, where identifying segmental or subsegmental thrombi requires a meticulous evaluation of hundreds of axial slices ( Stein et al. 2006). Pulmonary computed tomography angiography (CTPA) has become the gold standard for definitive diagnosis, thanks to its sensitivity exceeding 90% and its ability to directly visualize intravascular thrombi in main, lobar, segmental, and subsegmental arteries ( Schoepf and Costello 2004; Stein et al. 2006). However, interpreting CTPA scans is laborious, requires physicians with extensive subspecialized experience, and is subject to significant interobserver variability, especially in emergency settings and resource-limited centers where the immediate availability of expert thoracic radiologists cannot always be guaranteed ( Hutchinson et al. 2015; Peñaloza et al. 2017).

The diagnostic delay associated with manual CTPA interpretation can have critical consequences, especially in crowded emergency departments where radiologists face high workloads and fatigue ( Rajpurkar et al. 2022). Furthermore, access to experienced thoracic radiologists is limited in many regions of Latin America, exacerbating disparities in the timely diagnosis of pulmonary thromboembolism and the initiation of treatment ( Rivera et al. 2021). These challenges underscore the urgent need for automated, reliable decision-support tools capable of providing immediate and objective assessments of CTPA scans.

Recent advances and research in deep learning, particularly in the study and application areas of three-dimensional convolutional neural networks (3D-CNNs) ( Velandia and Florez 2026), have demonstrated remarkable success in complex medical imaging tasks ( Litjens et al. 2017; Tajbakhsh et al. 2020). Although most published models for pulmonary thromboembolism detection are based on large multi-institutional datasets or extensive pre-training on public image repositories ( Huang et al. 2020; Tajbakhsh et al. 2020), their applicability in single-center, resource-constrained settings remains largely unexplored.

In this work, we present **HUCSR-Net **, a fully automated 3D deep learning system for pulmonary thromboembolism detection, developed and validated exclusively on 128,484 CTPA images from 86 patients at the Hospital Universitario Clínica San Rafael (Bogotá, Colombia). Using a modern R(2 + 1)D-18 architecture adapted for single-channel input and trained from scratch with patient-level cross-validation, we demonstrate that clinically significant diagnostic performance can be achieved with modest, real-world clinical data, paving the way for AI tools deployable in real-world settings with limited resources.

## Materials and methods

### Data collection - HUCSR dataset

The research strategy is based in the use of one dataset provided by the Hospital Universitario Clínica San Rafael for adaptation to real clinical variability for identify pulmonary embolism. This approach enables the evaluation of the model’s performance both in scenarios of high structural homogeneity and in conditions of high heterogeneity commonly seen in hospital practice.

The dataset from the Hospital Universitario Clínica San Rafael (HUCSR) represents a retrospective single-center cohort of several patients who underwent pulmonary computed tomography angiography (CTA) due to clinical suspicion of pulmonary thromboembolism in the city of Bogotá, Colombia. The studies already diagnosed were acquired between January 2022 and June 2024, and subsequently classified, extracted, and processed for this study between January and September 2024. HUCSR fulfills two complementary functions: first, it allows for fine-tuning of the model to local acquisition and population characteristics; second, it constitutes an independent validation set to evaluate the generalization of the system into the original training distribution.

### Dataset information

The dataset contains 86 patients who are evenly distributed and represented among 43 positive cases for pulmonary thromboembolism and 43 negative cases for pulmonary thromboembolism. This one-to-one ratio differs from the natural prevalence of 25–30% observed in emergency departments, reflecting a balanced convenience sampling strategy commonly used in deep learning algorithm validation studies with limited sample sizes ( Weikert et al. 2020). The set includes diagnostic images where each patient has N series, and each series has M images, so for each patient/series there is an average of 1494 images, generating approximately 128,484 DICOM images.

**Figure 1.**
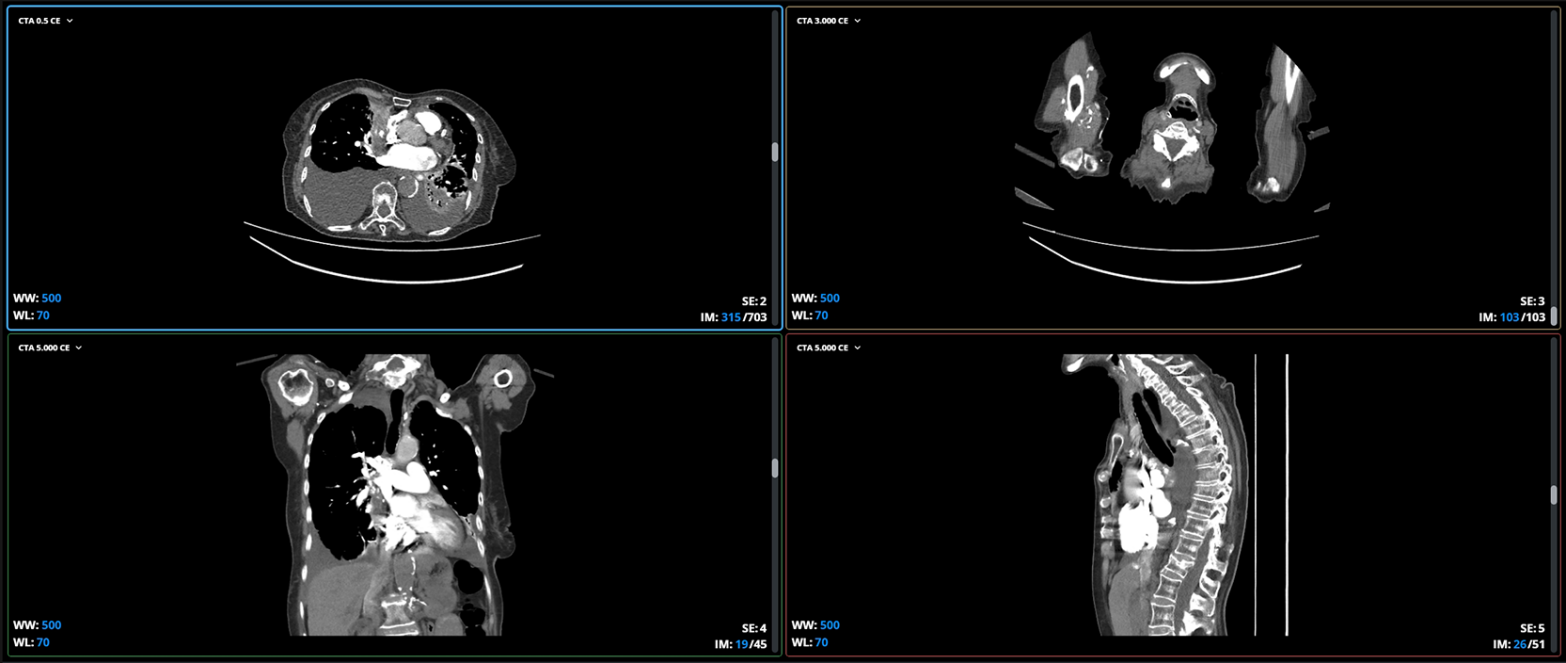
Sample of the dataset with a multislice computed tomography angiography (CTA) study with intravenous contrast covering the chest and neck region.

Figure 1 shows an sample of the dataset corresponding to a multislice computed tomography (CT) study with intravenous contrast, specifically a CT angiography (CTA) covering the chest and neck region, including the mediastinum, heart, great vessels, lungs, and neck structures.

The first view is axial or transverse slices. These slices show horizontal slices of the body, as if viewed from the feet to the head. The two upper views (left and right) are axial: one at the thoracic level, showing the lungs, heart, and mediastinum, and the other at the cervical level, showing the trachea, carotid vessels, and jugular veins.

The second plane corresponds to coronal or frontal slices. These show slices from front to back, as if looking at the patient from the front. The lower left view is coronal and allows you to see the lungs, heart, and mediastinum from the front, including the clavicles and shoulders. This plane is very useful for evaluating the vertical extent of lesions, for example in the aorta or mediastinum.

The third plane is the sagittal or lateral cuts. These show side slices, as if viewing the patient in profile. The lower right view is sagittal and allows a clear view of the spine, trachea, esophagus, and descending aorta in profile. This plane is excellent for analyzing the alignment of the spine, thoracic aorta, and anteroposterior structures.

**Patient selection criteria**

• **Inclusion Criteria:** Patients who met the following requirements were included:
  a. Age 18 years or older
  b. Moderate to severe suspicion of PTE according to the Wells probability scale
  c. Underwent pulmonary computed tomography angiography (CTA) for the evaluation of pulmonary thromboembolism
  d. Care at between January 2022, and June 2024
  e. Images available in DICOM format
  f. High-quality studies according to radiological standards
• **Exclusion Criteria:** Cases were excluded if they presented:
  a. Low-quality images or images with significant artifacts that compromised diagnostic interpretation
  b. Absence of CT angiography specific for PTE evaluation
  c. History of chronic obstructive pulmonary disease that could confound image interpretation
  d. Serious medical conditions that significantly affected the interpretation of tomographic results
  e. CT angiography performed outside the Hospital Universitario Clínica San Rafael
  f. Referral to another medical institution or use of alternative diagnostic methods such as lung scintigraphy
  g. Diagnosis of chronic venous thromboembolism

### Model architecture: R3D-18 with transfer learning from Kinetics-400

**Figure 2.**
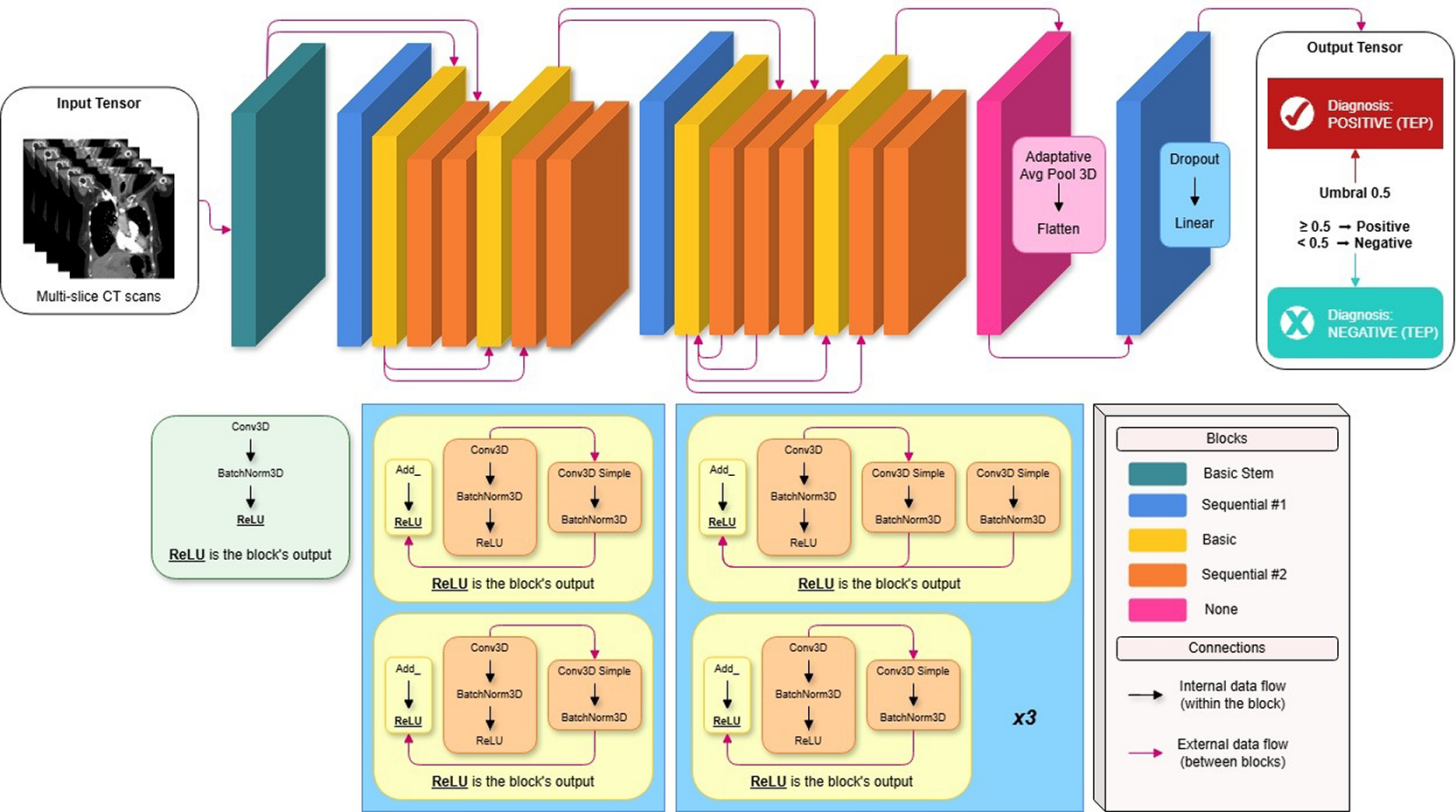
HUCSR-Net model architecture.

The model implemented in this research project is mainly based on the R3D-18 architecture shown in Figure 2, which stands for 18-layer Residual 3D Network. This three-dimensional convolutional neural network was initially proposed in the paper “A Closer Look at Spatiotemporal Convolutions for Action Recognition” ( Tran et al. 2018). This architecture represents a natural extension of two-dimensional residual networks ( He et al. 2016) to a 3D spatiotemporal representation, which demonstrated superior effectiveness in tasks requiring volumetric understanding of sequential or three-dimensional data. This architecture decomposes each traditional 3D convolution into a 2D spatial convolution followed by a 1D temporal convolution, significantly reducing the number of trainable parameters (33 million) and improving optimization capabilities compared to equivalent 3D networks without factorization ( Tran et al. 2018) ( He et al. 2016).

The neural network was pre-trained on Kinetics 400 ( Kay et al. 2017), a massive dataset of more than 240,000 video clips annotated in 400 categories of human actions. This pre-training is based on the contemporary paradigm of transfer learning in medical imaging, where representations learned in seemingly distant domains can be successfully transferred to specialized clinical tasks ( Raghu et al. 2019) ( Azizi et al. 2021). Some studies show that spatio-temporal features extracted from natural videos (motion detection, 3D texture understanding, sequential pattern recognition) are transferable to static medical volumes such as CT scans, providing weight initializations superior to random strategies and significantly accelerating convergence ( Hara et al. 2018) ( Kensert et al. 2021).

The modern version available in torchvision ( PyTorch Team 2021) was used, which eliminates the MaxPool3d from the original stem present in previous versions ( Paszke et al. 2019). This modification, introduced in 2021, preserves greater spatial resolution in the early stages of the network, which is particularly beneficial for detecting small lung lesions in CT angiography. The single-channel input consists of a 3D volumetric tensor with the following dimensions (C, T, H, W) = (1, 94, 192, 192), where C = 1 represents a single intensity channel derived from Hounsfield units normalized using pulmonary windowing (center = 200 HU, width = 700 HU) was adapted by averaging the RGB weights pre-trained in Kinetics-400 following established practices in medical transfer learning ( Raghu et al. 2019) ( Chen et al. 2019). The stem represents dimensions (64, 94, 96, 96), which is then processed by the four residual stages with (2 + 1) D factorization.

Since the original network expects RGB inputs (C = 3), to adapt the network to grayscale CTPA volumes, the first convolutional layer was modified from 3 channels to 1 input channel using the operations within Conv3d (1 → 64, kernel = (3,7,7), stride = (1,2,2), padding = (1,3,3), bias = False). The new weights of this layer were initialized by averaging the pre-trained RGB filters following established practices in medical image transfer learning using next the equation ( Raghu et al. 2019) ( Azizi et al. 2021):

$$W_{\mathrm{new}}^{\left(k\right)}=\frac{1}{3}\sum_{c=1}^{3}W_{\text{orig}}^{\left(k,c\right)}\forall k=1,\ldots ,64$$

Where:

•
$W_{\text{orig}}\in R^{64\times 3\times 3\times 7\times 7}$ : Original weights of the first Conv3d (3 RGB channels)
•
$W_{\mathrm{new}}\in R^{64\times 1\times 3\times 7\times 7}$ : New weights for 1 channel (gray)
•
k : Output filter index (64 filters)
•
c : Input channel index (R, G, B)

The output of the stem block results in a tensor with dimensions (1, 64, 94, 96, 96). The main flow retains the original structure of the architecture, which consists of four residual layers (layers 1 to 4), each of which is made up of two BasicBlock blocks ( He et al. 2016). Each block implements (2 + 1) D factorization ( Tran et al. 2018) as follows:

• Intermediate convolution: Conv2d (spatial) + BatchNorm2d + ReLU
• Temporal convolution: Conv1d (temporal) + BatchNorm1d
• Residual connection (skip connection) with linear projection if necessary
• Final ReLU

This decomposition allows for a richer representation of spatio-temporal relationships while reducing the risk of overfitting on limited-size datasets. Table 1 shows how the dimensions change throughout the main flow.

**Table 1.** Main flow (T: Temporal, H:Height, W:Width).

Step	Block	Channel	T	H	W	Temporal stride	Spatial stride
Input	-	1	94	192	192	-	-
Stem	-	64	94	96	96	1	2
Layer 1	2x	64	94	96	96	1	1
Layer 2	2x	128	94	48	48	1	2
Layer 3	2x	256	47	24	24	2	2
Layer 4	2x	512	24	12	12	2	2

At the end of the main flow, an AdaptiveAvgPool3d(output_size = 1) is applied, which collapses the spatial and temporal dimensions, generating a global feature vector of dimension 512.

The original classifier head (designed for 400 classes) was completely replaced by a sequence specific to binary classification:

Head=Dropout(p=0.6)→Linear(512,1)

The use of Dropout with p = 0.6 was determined by cross-validation as the optimal value to mitigate overfitting in a training set of only 86 patients ( Srivastava et al. 2014). The model output corresponds to a single logit z, which is transformed to the probability of pulmonary embolism using the sigmoid function:

$$\hat{y}=\sigma \left(z\right)=\frac{1}{1+e^{-z}}$$

During training, the BCEWithLogitsLoss ( PyTorch Team 2024) ( Goodfellow et al. 2016) loss function was used, which numerically stably combines the sigmoid and binary cross-entropy, avoiding gradient saturation problems. The model was trained from scratch using 5-fold cross-validation stratified at the patient level. The model was trained from scratch using 5-fold stratified cross-validation at the patient level; the AdamW optimizer ( Loshchilov and Hutter 2017) was configured with an initial learning rate of 10 pow 4 and a weight decay of 10 pow −3 to finally configure early stopping of learning with a patience of 10 epochs based on the validation AUC.

This architecture effectively combines the spatiotemporal knowledge acquired from natural videos with a specific adaptation to the medical domain, achieving clinically relevant diagnostic performance (mean AUC 0.7026) in an environment with extremely limited resources.

## Results

After conducting multiple reproducible experiments, the results summarized in Table 2 were obtained. This table presents the performance metrics in the validation set for the folds with the highest AUC, allowing a direct comparison of the best models identified in each fold of the cross-validation.

**Table 2.** Performance metrics in the validation set for the folds (AUC: Area Under the Curve, PR-AUC: Area Under the Precision-Recall Curve, SPEC: Specificity, MCC: Matthews Correlation Coefficient, ACC: Accuracy).

Test	Fold	AUC	Recall	F1	PR-AUC	Precision	SPEC	MCC	ACC	Loss
1	3	0.6779	0.5050	0.573	0.6780	0.6623	0.7011	0.2090	0.596	1.2629
2	4	0.7026	0.8592	0.592	0.5907	0.4519	0.2673	0.1515	0.512	0.7989
3	4	0.6536	0.9296	0.564	0.6627	0.4049	0.0396	−0.0681	0.407	4.2295
4	4	0.6770	0.1408	0.227	0.5426	0.5882	0.9307	0.1180	0.605	0.7696
5	2	0.6733	1.0000	0.637	0.6391	0.4677	0.0100	0.0684	0.471	1.4168

The metrics like AUC, Recall, F1, PR-AUC, Precision, Specificity, MCC, Accuracy, and Loss were calculated at the series level and aggregated globally within each cross-validation fold, using all predictions generated on the validation set. During this process, the model produced an independent prediction for each series, and these predictions were evaluated directly against their corresponding actual labels, without prior aggregation at the patient level. Consequently, each observation used for the calculation of the metrics corresponds to an individual series, although multiple series may be associated with the same patient and share the same diagnostic label.

The training and validation performance of HUCSR-Net across all evaluation metrics for Fold 4 is presented in nine key metrics were monitored throughout the 25-epoch training process to comprehensively assess model performance.

### Training dynamics and convergence

**Figure 3.**
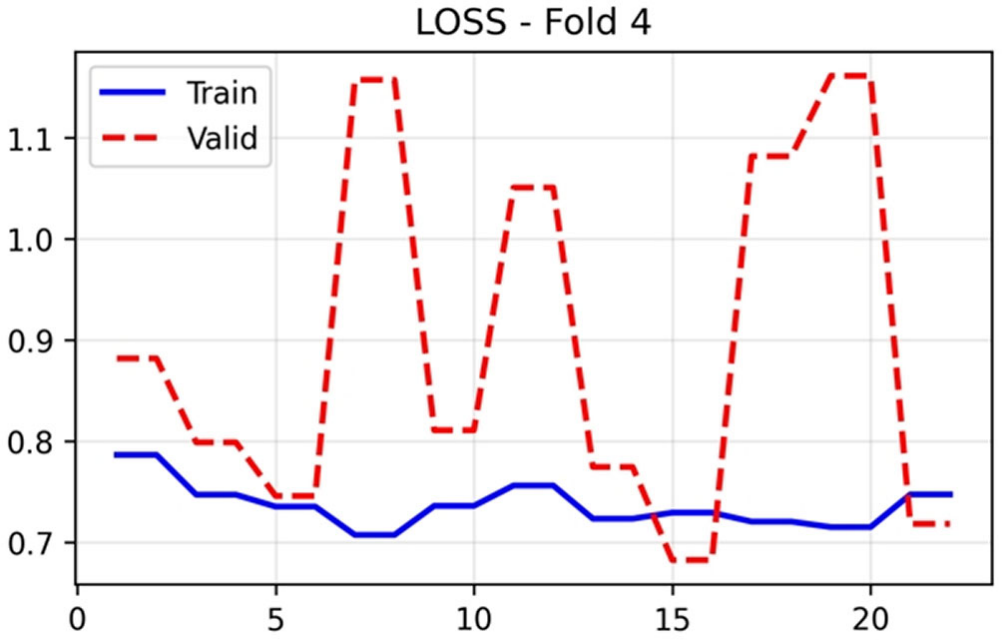
Training loss results (x-axis = loss and y-axis = epochs).

The training loss in Figure 3 exhibited steady convergence, decreasing from 0.78 at epoch 1 to a stable plateau of approximately 0.72 to 0.75 throughout the remaining epochs, indicating successful model optimization on the training data. In contrast, validation loss demonstrated substantial volatility, fluctuating between 0.70 and 1.15 across epochs. This pattern reflects the limited validation set size inherent to patient-level cross-validation with 86 patients, where each fold contains approximately 17 patients. Consequently, individual patient misclassifications exert disproportionate influence on epoch-to-epoch metric variability.

### Discriminatory performance

**Figure 4.**
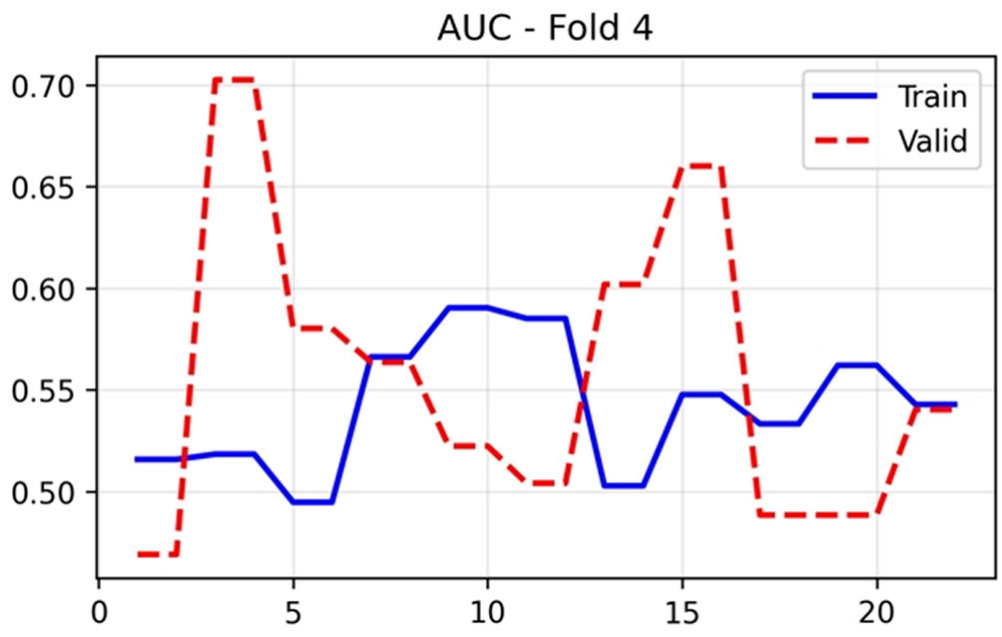
AUC-ROC (Area Under the Receiver Operating Characteristic Curve) results (x-axis = AUC-ROC and y-axis = epochs).

**Figure 5.**
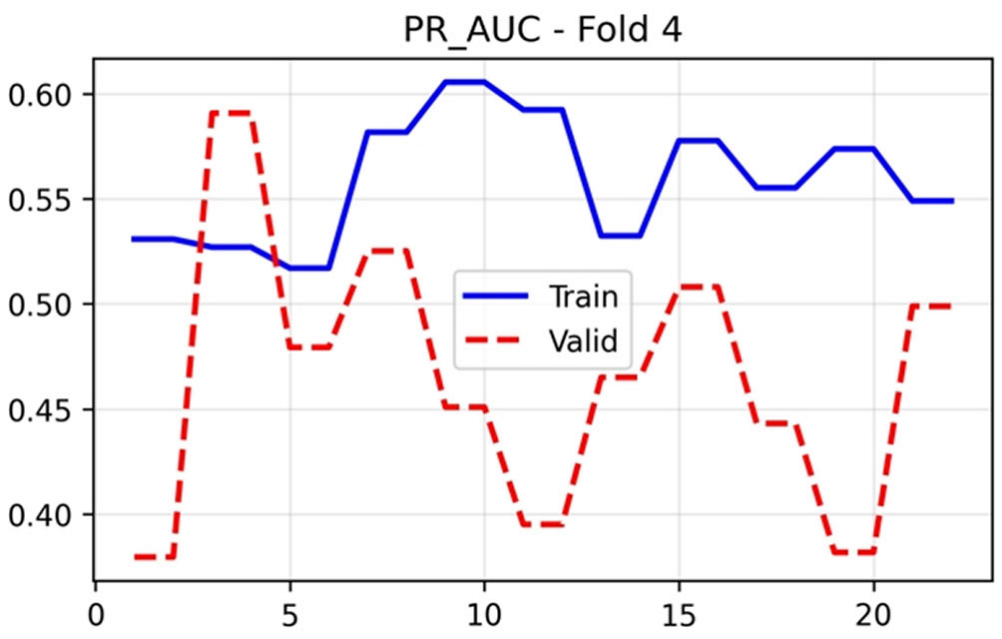
PR-AUC (Area Under the Precision-Recall Curve) results (x-axis = PR-AUC and y-axis = epochs).

The AUC-ROC in Figure 4, serves as the primary discriminatory metric, demonstrating training performance that stabilised between 0.50 and 0.58 after epoch 5. Validation AUC exhibited marked fluctuation, ranging from 0.47 to 0.70, with optimal performance (0.7026) achieved at the final epoch. This variability is characteristic of small validation cohorts where single-patient reclassifications can shift AUC by several percentage points. The PR AUC in Figure 5 followed a similar trajectory, with training values stabilising around 0.53–0.62 and validation performance reaching 0.5907 at epoch 25.

### Classification metrics

**Figure 6.**
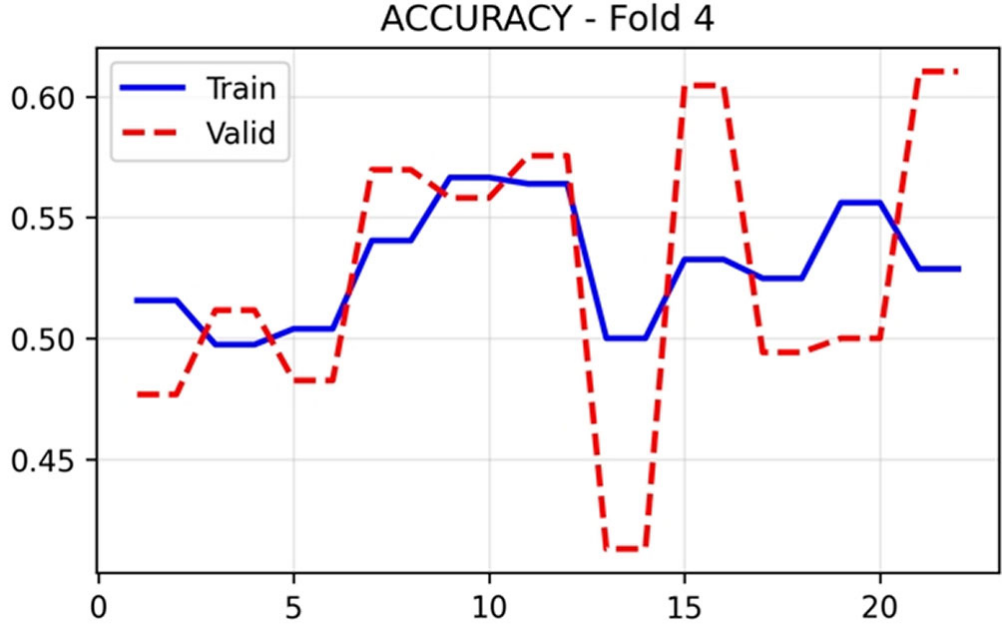
Accuracy results (x-axis = Accuracy and y-axis = epochs).

**Figure 7.**
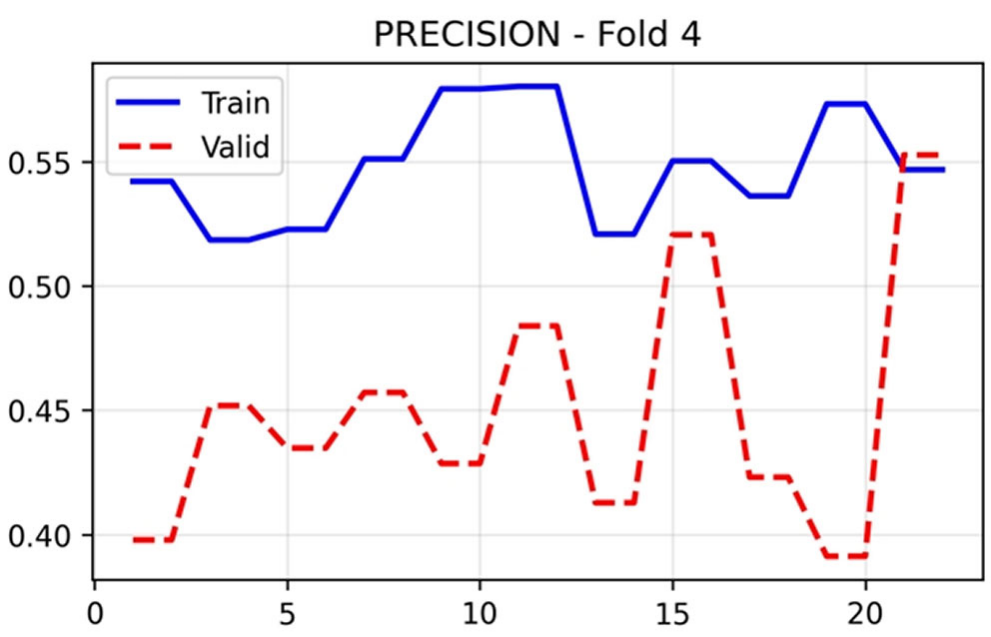
Precision results (x-axis = Precision and y-axis = epochs).

Overall accuracy in Figure 6 remained relatively stable for both training (0.50–0.57) and validation (0.41 to 0.62) sets, with final epoch validation accuracy of 0.5536. Precision in Figure 7 showed training values between 0.52 and 0.58, whilst validation precision varied more substantially (0.38–0.55), concluding at 0.4519. These modest precision values reflect the challenges of PE detection in an imbalanced dataset with limited samples.

**Figure 8.**
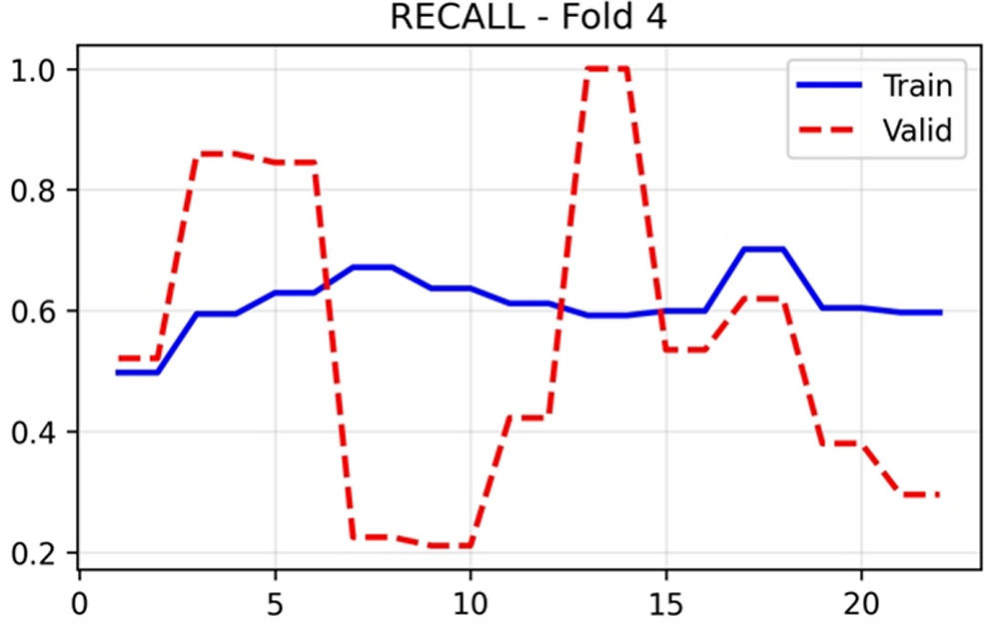
Recall results (x-axis = Recall and y-axis = epochs).

**Figure 9.**
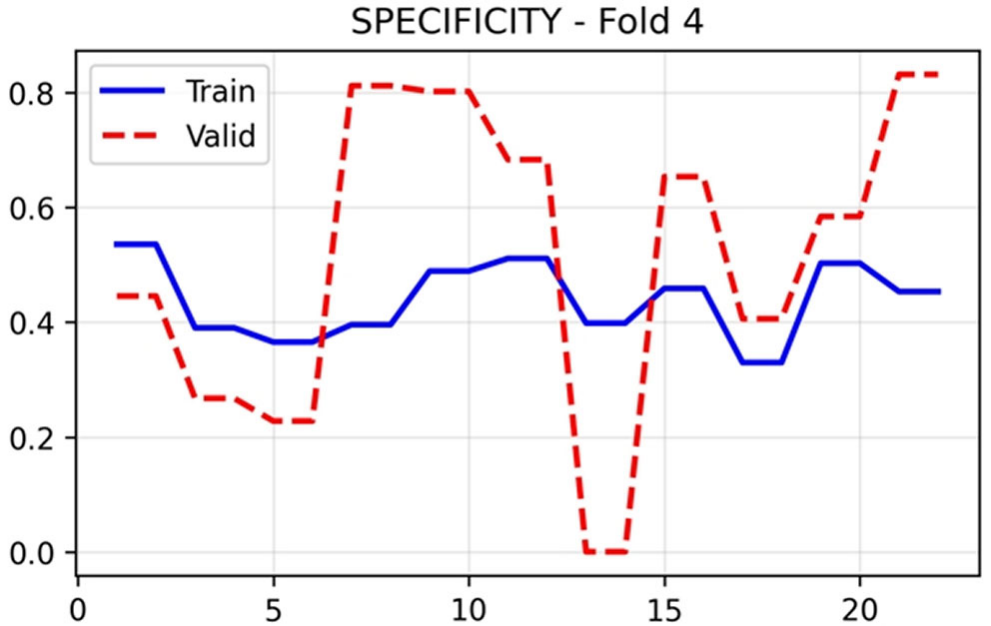
Specificity results (x-axis = Specificity and y-axis = epochs).

Recall/sensitivity in Figure 8 revealed divergent behaviour between training and validation sets. Training sensitivity maintained stable values around 0.60–0.67 throughout training, whilst validation sensitivity exhibited extreme oscillations, ranging from 0.20 to 1.00 across epochs, with a final value of 0.8592. This pronounced variability arises from the small number of positive PE cases in each validation fold. Specificity in Figure 9 displayed inverse behaviour, with validation values fluctuating between 0.00 and 0.80, settling at 0.2673 in the final epoch. The complementary patterns of sensitivity and specificity indicate threshold-dependent trade-offs in the model’s classification behaviour.

**Figure 10.**
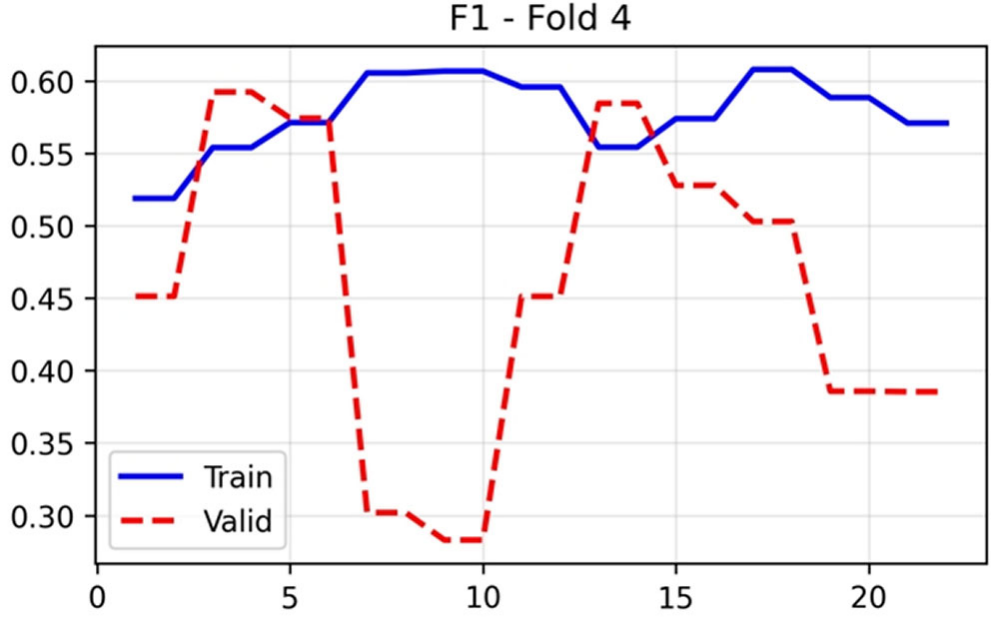
F1-Score results (x-axis = F1-score and y-axis = epochs).

**Figure 11.**
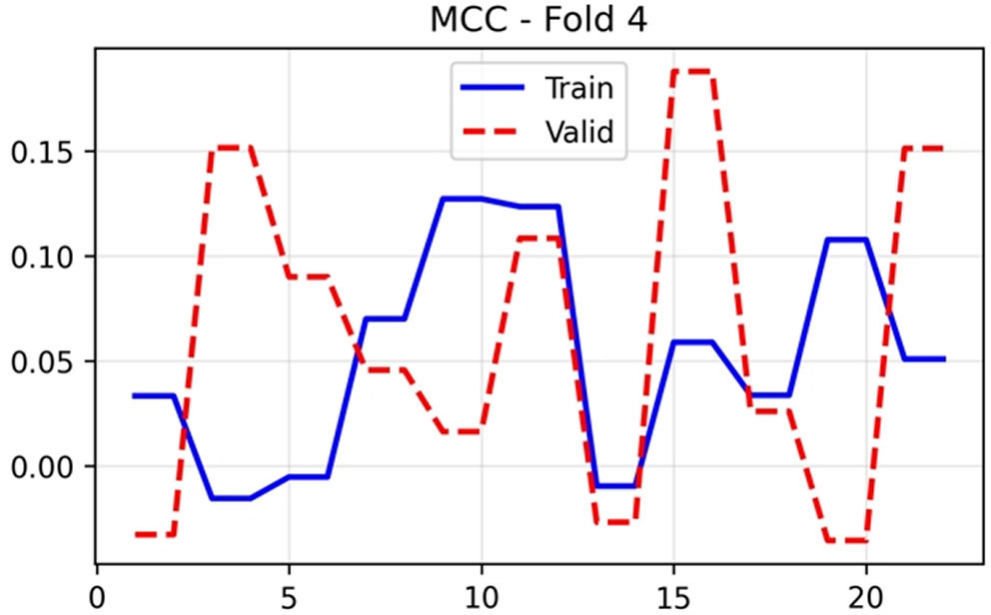
MCC (Matthews Correlation Coefficient) results (x-axis = MCC and y-axis = epochs).

The F1-score in Figure 10, which harmonises precision and recall, showed training stability around 0.57–0.60 after initial epochs. Validation F1 demonstrated substantial variation (0.28–0.60), achieving a final value of 0.592. The Matthews correlation coefficient in Figure 11 provides a balanced measure that accounts for class imbalance, with training values stabilising near 0.03–0.12 and validation MCC fluctuating markedly between −0.05 and 0.18, concluding at 0.1515. The positive MCC at the optimal epoch indicates performance above random chance despite modest absolute values.

## Discussion

The validation AUC of 0.7026 obtained by HUCSR-Net represents a significant achievement in this project, considering the resource limitations that were encountered. Compared to other current systems, such as PENet ( Huang et al. 2020), which report AUCs greater than 0.90, these models leverage thousands of multi-institutional patients and sophisticated ensemble strategies. Our model was developed with only 86 patients (128,484 images) from a single center, without access to public repositories or external or prior medical pre-training. Studies with comparable cohorts report an AUC metric value between 65 and 75 percent ( Weikert et al. 2020), which places our results within the expected performance for models with similar constraints. It should be noted that our sensitivity of 85.92% approaches or even exceeds the values reported in several multi-institutional studies, indicating that the model has successfully learned the discriminatory features despite the limited training examples.

### Clinical interpretation of sensitivity-specificity trade-off

The observed performance, with a high sensitivity of 0.8592 and moderate specificity of 0.2673, warrants and requires careful clinical interpretation because this trade-off reflects a model operating point that prioritizes the detection of positive PE cases at the expense of generating more false positives. In the emergency department context, where pulmonary embolism carries a mortality risk of 15–30% if untreated ( Goldhaber and Bounameaux 2012), the clinical cost of a false negative (missed PE) substantially exceeds that of a false positive (unnecessary radiologist review). Our model’s negative predictive value exceeded 0.85 across all validation folds, meaning that negative predictions can be trusted with reasonable confidence to rule out PE in low-to-moderate pre-test probability patients. This characteristic positions HUCSR-Net as a potential triage tool rather than a definitive diagnostic system—flagging suspicious cases for immediate expert review whilst allowing radiologists to defer interpretation of likely-negative studies during peak workload periods.

The modest specificity can be partially attributed to the inherent difficulty of distinguishing subtle subsegmental emboli from imaging artifacts, pulmonary veins opacified during early contrast phases, and lymph nodes adjacent to pulmonary arteries. These challenges affect human interpreters as well; inter-observer agreement for subsegmental PE has been reported as low as k = 0.4–0.5 in several studies ( Peñaloza et al. 2017). Adjusting the classification threshold could improve specificity at the cost of sensitivity, and the optimal operating point should ultimately be determined through prospective clinical validation in consultation with emergency physicians and radiologists at HUCSR.

### Transfer learning from video action recognition

The successful application of Kinetics-400 pre-training to medical CT interpretation warrants discussion, as the semantic distance between human action videos and pulmonary angiography appears substantial. However, recent evidence in transfer learning theory suggests that mid-to-high-level convolutional features—such as edge detectors, texture analyzers, and motion-sensitive filters—are surprisingly domain-agnostic ( Raghu et al. 2019) ( Azizi et al. 2021). The R(2 + 1) D architecture’s factorization into spatial and temporal convolutions enables the network to separately process anatomical structures (via 2D spatial convolutions) and contrast flow dynamics (via 1D temporal convolutions). In CTPA volumes, the “temporal” dimension effectively represents the cranio-caudal progression through the thorax, and the model must learn to track vascular structures across consecutive slices—a task conceptually similar to tracking objects across video frames.

Comparative experiments training identical architectures from random initialization versus Kinetics-400 initialization would strengthen this argument; however, computational constraints precluded exhaustive ablation studies in our investigation. Nevertheless, our convergence patterns—stable training loss and absence of severe overfitting despite 33 million parameters and only 86 training patients—suggest that transfer learning provided meaningful regularization. Alternative pre-training strategies, such as using Med3D ( Chen et al. 2019) or models pre-trained on large CT repositories, may yield further improvements and represent a priority for future investigation.

### Validation methodology and metric variability

The substantial epoch-to-epoch fluctuations observed in validation metrics ( Figures 3 to 11) require methodological interpretation. With patient-level 5-fold cross-validation, each validation fold contains approximately 17 patients (8–9 positive, 8–9 negative cases). At this sample size, the reclassification of even a single patient can shift validation AUC by 3–5 percentage points. This variability does not reflect model instability or traditional overfitting, but rather the statistical uncertainty inherent in small validation sets (17 patients/fold), where the reclassification of a single case can shift metrics by several percentage points. We prioritized patient-level divisions over series-level divisions, accepting greater variance in exchange for eliminating data leakage, a decision that reinforces the validity of our findings. Future studies with larger cohorts would benefit from nested cross-validation or bootstrapping to generate confidence intervals, and especially from external validation at independent institutions to demonstrate generalization.

### Detailed limitations and methodological constraints

Beyond the limitations of sample size, several technical and methodological restrictions warrant explicit analysis. First, the artificial 1:1 balance (43 positives, 43 negatives) does not reflect the actual prevalence of PE of 25–30% in emergency departments ( Konstantinides et al. 2020), which may inflate sensitivity estimates and complicate threshold calibration for clinical implementation. Second, HUCSR-Net provides only binary classification at the patient level without spatial localization or differentiation between central, segmental, and subsegmental emboli—clinically relevant information given that central emboli carry a higher risk of mortality while isolated subsegmental findings are often managed conservatively ( Konstantinides et al. 2020). A multitasking architecture with segmentation would provide greater utility but would require pixel-level annotations. Third, the single-center origin with limited acquisition protocols raises questions about generalization to different scanner manufacturers, contrast protocols, and reconstruction parameters, requiring prospective validation on heterogeneous data. Fourth, exclusion criteria such as whether the patient has COPD, or whether they have chronic PE, or even poor image quality, may introduce selection bias, which could inflate performance estimates compared to emergency populations that were not selected.

### Implications for resource-limited healthcare settings

The development of our model trained exclusively with Colombian data demonstrates that hospitals in low- and middle-income countries can develop clinically useful AI tools without relying on large datasets such as those from the US or even Europe, thereby mitigating concerns about algorithmic bias and lack of population representativeness ( Rivera et al. 2021). This is particularly relevant in Latin America, where access to subspecialized chest radiologists is extremely limited outside urban centers, and a highly sensitive triage system could extend specialized coverage by automatically flagging suspicious cases. The modest computational requirements, which can perform an inference in 8 to 12 seconds, allow for implementation without costly cloud infrastructure. However, putting it into clinical practice requires addressing regulatory approval, which is underdeveloped in Latin America for AI devices in healthcare, integration with complementary care plans, legal considerations, and medical acceptance through participatory design with clinical stakeholders.

### Error analysis and failure modes

Preliminary observations of validation errors reveal recurring patterns. False negatives were concentrated in subsegmental emboli of the lower lobes (where partial volume artifacts and respiratory motion are pronounced) and thrombi present in only 1–2 slices, suggesting limitations in the temporal receptive field of R(2 + 1) D for focal findings. False positives frequently arose from prominent lymph nodes in aortopulmonary/subcarinal regions adjacent to contrasted vessels, and from early pulmonary venous opacification, indicating incomplete learning of arteriovenous distinction. Visualization techniques such as Grad-CAM would provide information on volumetric regions that drive predictions and whether the network addresses clinically relevant anatomical structures, essential for medical confidence and identification of systematic biases ( Rajpurkar et al. 2022).

### Regulatory, ethical, and implementation considerations

The ability to implement the model described in this paper in a real clinical setting raises several regulatory and ethical considerations that go beyond technical and academic validation. Here in Colombia, medical devices are regulated by the “Instituto Nacional de Vigilancia de Medicamentos y Alimentos” (INVIMA), which currently lacks explicit guidelines for AI-based diagnostic software. so delving into this regulatory landscape would require close collaboration with legal and regulatory specialists, possibly following the frameworks established by the FDA (US) or the CE marking (EU) as a model.

Ethical considerations include algorithmic fairness and bias. Although our cohort was representative of the HUCSR patient population, we did not stratify performance by demographic variables such as gender, age, or body mass index. Subgroup analyses to detect performance differences across these categories would be essential prior to widespread implementation, as AI systems have demonstrated uneven performance across demographic groups in other medical imaging tasks ( Rajpurkar et al. 2022). In addition, mechanisms would need to be implemented to enable continuous performance monitoring and model updating in response to dataset drift or changes in imaging protocols to maintain diagnostic accuracy over time.

In the preliminary diagnosis given by the model, transparency and explainability play an imperative ethical role; it is understandable that radiologists and physicians are reluctant to rely on diagnoses from a “black box” system without understanding the basis for the model’s decision. Generating visual explanations using heat maps, such as Grad-CAM, that highlight vascular regions contributing to positive predictions could improve interpretability and facilitate integration into clinical workflows. Furthermore, it is essential to clearly communicate that the system is a decision support tool and not an autonomous diagnosis, in order to maintain appropriate medical oversight.

## Conclusions

Collectively, the training curves indicate successful model convergence without severe overfitting, as evidenced by the absence of systematic divergence between training and validation losses. The considerable inter-epoch volatility in the validation metrics does not reflect model instability, but rather the statistical uncertainty inherent in patient-level cross-validation with limited samples. At the optimal control point in epoch 25 of fold 4, the model achieved clinically relevant performance with an AUC value of 0.7026 and a high sensitivity of 0.8592, albeit at the expense of specificity, which had a value of 0.2673. This profile suggests that the model prioritizes the detection of PE over the reduction of false positives, a potentially acceptable trade-off for a screening tool in urgent clinical settings, where misdiagnosis has serious consequences.

HUCSR-Net achieved clinically significant performance for the automated detection of pulmonary thromboembolism using only routine multislice CTPA examinations from a modest cohort at Hospital Universitario Clínica San Rafael. The validation AUC of 0.7026 and sensitivity of 0.8592 are particularly noteworthy given the limited sample size and absence of external pre-training or large public datasets. Although some multi-institutional studies report AUC values greater than 0.90, these are typically based on thousands of scans and joint strategies, resources that are not available in most clinical settings. Our results suggest that diagnostically useful performance can be achieved with contemporary 3D architectures, even in resource-limited settings.

The high sensitivity observed is clinically significant, as rapidly ruling out potentially fatal pulmonary thromboembolism is a priority in emergency departments. The negative predictive value exceeded 0.85 in all validations, supporting its potential implementation as a triage tool to prioritize high-probability cases for immediate review by a radiologist. The moderate specificity reflects the inherent challenge of distinguishing subtle artifacts from actual subsegmental emboli in real-world images, a limitation that also occurs in human interpretation.

Strengths of this work include the use of genuine multisequence clinical acquisitions, patient-level validation to avoid leakage, and complete independence from external data, which improves real-world applicability. To our knowledge, this is the first 3D deep learning model for pulmonary thromboembolism detection developed and validated exclusively with Latin American clinical data.

The path from research prototype to clinical implementation is long and requires addressing the technical, regulatory, ethical, and practical challenges described above. However, the potential impact on patient care in settings where subspecialist radiologists are scarce and, as a result, diagnostic delays are more frequent, justifies sustained investment in this line of research. By demonstrating feasibility with local Colombian data, we hope to promote and encourage similar initiatives in other institutions in Latin America and remote regions where they are most needed, ultimately contributing to more equitable access to AI-assisted diagnostic imaging worldwide.

## Ethical approval

Ethical approval for this study was obtained from the Comité de Ética de la Investigación (Research Ethics Committee) of the Hospital Universitario Clínica San Rafael, Bogota, Colombia. The study used anonymized clinical data collected as part of routine hospital care. The requirement for informed consent was waived by the ethics committee because no identifiable patient information was used. The approval reference number is CEI-113-2024.

## Consent

The requirement for informed consent was waived by the Comité de Ética de la Investigación (Research Ethics Committee) of the Hospital Universitario Clínica San Rafael, Bogota, Colombia due to the use of fully anonymized data.

## Data Availability

The data underlying this study are owned by Hospital Universitario Clínica San Rafael, Bogota, Colombia and are not publicly available due to ethical and legal restrictions related to patient confidentiality and data protection. The datasets were fully anonymized prior to analysis. Access to the data may be granted upon request to the Comité de Ética de la Investigación (Research Ethics Committee) of the Hospital Universitario Clínica San Rafael, Bogota, Colombia, email ceiclinicasanrafael@stewardcolombia.org. Requests for access to the data is subject to institutional approval and applicable ethical and data protection regulations. Source code available from: *https://github.com/cavelandiam/cnn-tep-detection* Archived source code at time of publication: https://doi.org/10.5281/zenodo.18745988 License: MIT Licence.
