# Supplementary material for: HUCSR-Net: Automated Pulmonary Embolism Detection Using 3D Deep Learning
Source: F1000Res. 2026 Apr 24;15:618. [Version 1] doi: 10.12688/f1000research.178627.1 (PMC13191312; doi:10.12688/f1000research.178627.1)
Supplement: Peer review history

# Reviewer report: Reviewer response for version 1

**DOI:** 10.5256/f1000research.197036.r481349

**Published:** 2026-05-20

**Authors:** Raphael de Aquino Gomes

**Affiliations:** 1 Instituto Federal de Goiás, Goiânia, Brazil

This manuscript presents HUCSR-Net, a 3D convolutional neural network for automated pulmonary embolism detection, trained on data from 86 patients at a single Colombian institution. The work addresses a genuine clinical need in resource-limited settings and represents a reasonable feasibility study. However, some issues must be resolved before acceptance.

 The most immediate concern is the use of the term "optimal model". In the mathematical sense, optimality implies the best achievable solution under a defined objective, a claim that cannot be supported by cross-validation or hyperparameter search alone. The authors should replace this language throughout with "best-performing model" or "selected configuration."

 A more fundamental problem is the insufficient framing of the data scarcity context in the abstract and introduction. The entire interpretive value of this work rests on the fact that the model was trained with only 86 patients, which naturally limits achievable performance and directly explains the low specificity and MCC. Neither the abstract nor the introduction makes this sufficiently clear, and no focused review of related small-cohort PE detection work is provided. The abstract's conclusion that the results are "comparable to published multi-thousand-patient studies" is not supported by the reported metrics and could mislead readers. The introduction should include a paragraph reviewing the literature on data-limited deep learning for PE detection, and the abstract should benchmark results against comparable small-data studies rather than against large multi-institutional models.

 On the editorial side, page 7 contains a duplicated sentence describing the 5-fold cross-validation setup. Additionally, all metrics in Table 2 are reported as point estimates from the single best fold, without confidence intervals or mean ± SD across folds, which introduces optimistic bias. The classification threshold used to derive binary sensitivity and specificity values is never stated, an omission critical for reproducibility.

 Minor issues include inconsistent alternation between "PE" and "PTE" without clarification, systematic axis label errors in the captions of Figures 3–11 (epochs and metric axes are inverted), and the absence of any mention in the abstract that the artificial 1:1 class balance differs from real-world PE prevalence of 25–30%. A general language editing pass is also recommended.

Are the conclusions about the tool and its performance adequately supported by the findings presented in the article?

Yes

Is the rationale for developing the new software tool clearly explained?

Yes

Is the description of the software tool technically sound?

Yes

Are sufficient details of the code, methods and analysis (if applicable) provided to allow replication of the software development and its use by others?

Yes

Is sufficient information provided to allow interpretation of the expected output datasets and any results generated using the tool?

Yes

Reviewer Expertise:

My research focuses on applying machine learning and deep learning methods to energy systems analysis, with an emphasis on distributed solar generation, photovoltaic forecasting, and the energy transition in Brazil.

I confirm that I have read this submission and believe that I have an appropriate level of expertise to confirm that it is of an acceptable scientific standard.

# Reviewer report: Reviewer response for version 1

**DOI:** 10.5256/f1000research.197036.r481351

**Published:** 2026-05-18

**Authors:** Kiran Puttegowda

**Affiliations:** 1 Vidyavardhaka College of Engineering Mysuru, karnataka, India

The manuscript titled **“HUCSR-Net: Automated Pulmonary Embolism Detection Using 3D Deep Learning”** presents a relevant, timely, and valuable contribution in the field of AI-assisted medical imaging, particularly for pulmonary embolism detection using 3D deep learning architectures. The study addresses an important clinical challenge and proposes a practical deep learning framework that is highly suitable for real-world and resource-constrained healthcare settings. The use of a modern R(2+1)D-18 architecture with transfer learning, patient-level 5-fold cross-validation, and clinically meaningful evaluation metrics demonstrates strong methodological rigor and scientific relevance. The reported validation performance (AUC of 0.7026 and sensitivity of 0.8592) is promising, especially considering the limited dataset size and the single-center cohort.

  

1. The authors should provide clearer justification for selecting the input volume dimensions (1, 94, 192, 192) and explain whether alternative input sizes were evaluated.
2. More details on threshold selection for sensitivity and specificity calculation would improve reproducibility and clarity.
3. Including confidence intervals for AUC, sensitivity, specificity, and F1-score would strengthen the statistical reliability of the reported results.
4. A brief comparative analysis between random initialization and Kinetics-400 pretraining would further support the effectiveness of the transfer learning strategy.
5. The discussion of false positives and false negatives could be strengthened by including representative case examples or visualization techniques such as Grad-CAM to improve interpretability.

Minor Non-Technical Revisions:

1. Minor grammatical issues and sentence structure inconsistencies are present throughout the manuscript and require careful language polishing.
2. Some sections in the Introduction and Discussion are slightly lengthy and could be made more concise to improve readability.
3. Figure captions should be made more descriptive and self-explanatory for better reader understanding.
4. Consistency in terminology should be maintained throughout the manuscript (e.g., pulmonary embolism vs pulmonary thromboembolism).
5. Reference formatting should be carefully reviewed to ensure uniform citation style across all references.

Overall, the work is novel, practically significant, and suitable for indexing. The study provides meaningful insights for AI-based pulmonary embolism detection and demonstrates strong potential for clinical decision-support applications in low-resource environments.

Are the conclusions about the tool and its performance adequately supported by the findings presented in the article?

Yes

Is the rationale for developing the new software tool clearly explained?

Yes

Is the description of the software tool technically sound?

Yes

Are sufficient details of the code, methods and analysis (if applicable) provided to allow replication of the software development and its use by others?

Yes

Is sufficient information provided to allow interpretation of the expected output datasets and any results generated using the tool?

Yes

Reviewer Expertise:

Medical image processing, signal processing

I confirm that I have read this submission and believe that I have an appropriate level of expertise to confirm that it is of an acceptable scientific standard.
